# Pneumatosis cystoides intestinalis: a case report and literature review

**DOI:** 10.1186/s12876-019-1087-9

**Published:** 2019-11-06

**Authors:** Fangmei Ling, Di Guo, Liangru Zhu

**Affiliations:** Division of Gastroenterology, Union Hospital, Tongji Medical College, Huazhong University of Science and Technology, No.1277, Jiefang Avenue, Wuhan, Hubei province China

**Keywords:** Pneumatosis cystoides intestinalis, Differential diagnosis, Treatment

## Abstract

**Background:**

Pneumatosis cystoides intestinalis (PCI) is a low-incidence disease that confuses many doctors. A vast number of factors are suspected to contribute to its pathogenesis, such as Crohn’s disease, intestinal stenosis, ulcerative colitis, drug use, extra-gastrointestinal diseases, and chronic obstructive pulmonary disease. Most consider its pathogenesis interrelated to an increase in intra-intestinal pressure and the accumulation of gas produced by aerogenic bacteria, and patients with atypical symptoms and imaging manifestations tend to be misdiagnosed.

**Case presentation:**

A 64-year-old man complained of a 3-month history of bloody stool without mucopurulent discharge, abdominal pain, or diarrhea. Colonoscopy revealed multiple nodular projections into the segmental mucosa of the sigmoid colon. Crohn’s disease and malignant disease ware suspected first according to the patient’s history, but laboratory examinations did not confirm either. Endoscopic ultrasound (EUS) revealed multiple cystic lesions in the submucosa. Moreover, computer tomography scan showed multiple bubble-like cysts. Combined with ultrasonography, computed tomography, and pathology findings, we ultimately made a diagnosis of PCI. Instead of surgery, we recommended conservative treatment consisting of endoscopy and oral drug administration. His symptoms improved with drug therapy after discharge, and no recurrence was noted on follow-up.

**Conclusions:**

The incidence of PCI is low. Due to a lack of specificity in clinical manifestations and endoscopic findings, it often misdiagnosed as intestinal polyps, tumors, inflammatory bowel disease, or other conditions. Colonoscopy, computed tomography, and ultrasonography have demonstrated benefit in patients with multiple nodular projections in colon. Compared to the treatment of the above diseases, PCI treatment is effective and convenient, and the prognosis is optimistic. Therefore, clinicians should increase their awareness of PCI to avoid unnecessary misdiagnosis.

## Background

Pneumatosis cystoides intestinalis (PCI) was first reported by Du Vernoi in 1730 [1]. In a systematic review and analysis of 239 patients with PCI, Wu et al. reported that the peak age at onset was 45.3 ± 15.6 years (range, 2–81 years), the male to female ratio was 2.4:1, and the mean disease course was 6 months [2]. After examining 123 patients with PCI, Boerner reported an equal incidence in male and female patients and that remission was achieved in 70% of patients using nonsurgical treatment [3].

PCI is distributed throughout the digestive tract, particularly the subserous or submucosa of the small intestine and colon, in which multiple pneumocysts develop. The distal stump of the transverse splenic flexure colon, particularly the descending and sigmoid colon, is most commonly affected [2].

## Case presentation

**Fig. 1:**
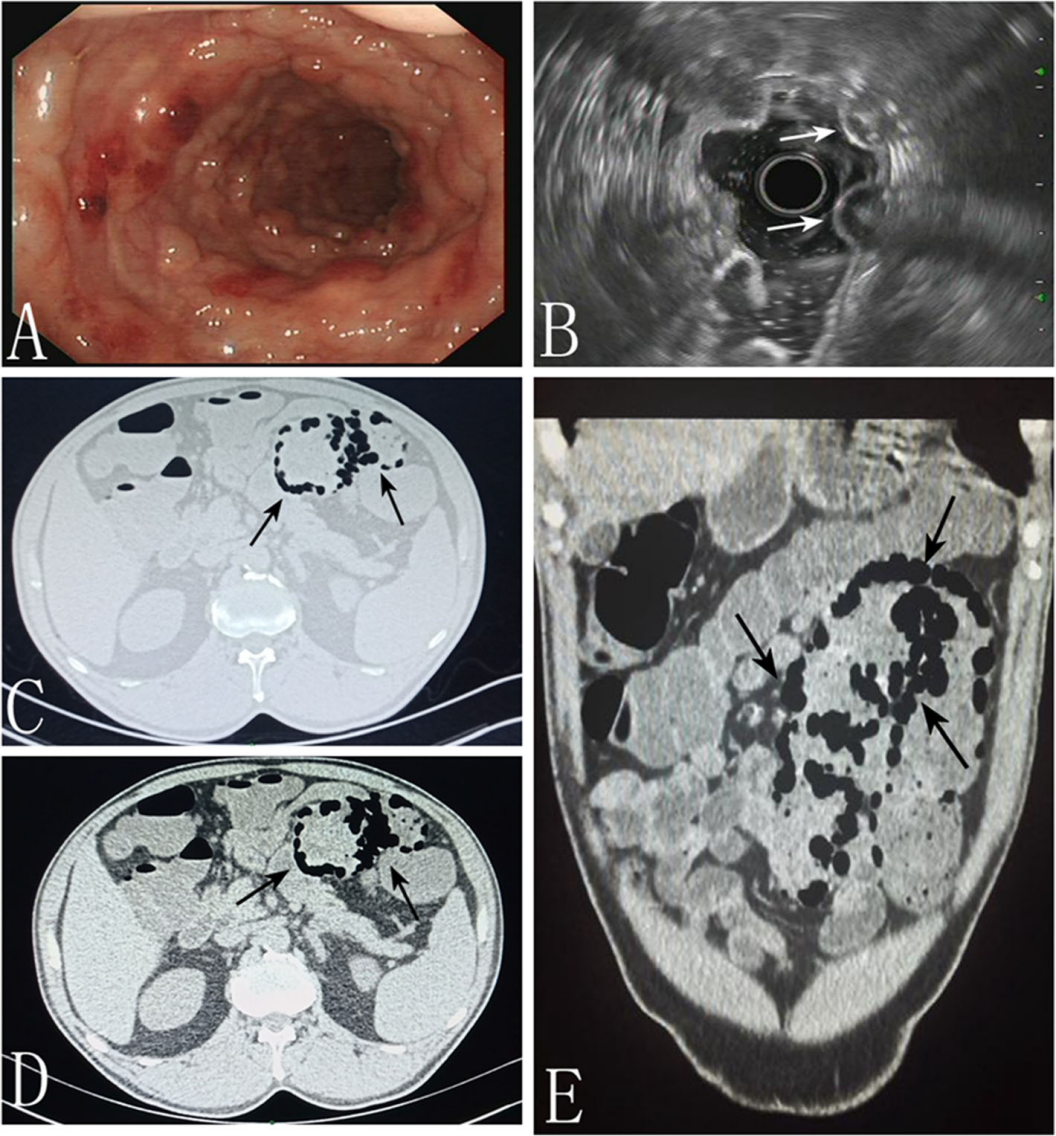
Endoscopic and CT examination. **a** Colonoscopy disclosed multiple nodular lesions in the transverse colon and sigmoid colon. **b** Endoscopic ultrasonography showed hypoechoic lesions (white allows). **c** CT Horizontal scan. Image adjusted to lung window (black allows). **d** CT Horizontal scan. Image adjusted to abdominal window (black allows). **e** CT Vertical scan. Multiple grape-like gases were visible in sigmoid colon ((black allows)

A 64-year-old man presented to the gastroenterology inpatient department with bloody stool in August 2017. There was no obvious cause of bloody stool before the patient was admitted to the hospital 3 months prior with mucus and blood attached to formed stools. The patient had no clear symptoms of abdominal pain, abdominal distension, tenesmus, fever, or night sweats. His weight had decreased by about 9 kg since he first became ill. He had a 5-year history of diabetes that was controlled by acarbose. A physical examination revealed no abnormalities. Laboratory investigations revealed no abnormalities except platelets count of 95 G/L. Colonoscopy revealed multiple nodular projections in the segmental mucosa of the sigmoid colon, some of which were transparent (Fig. 1a). Endoscopic ultrasound (EUS) revealed multiple cystic lesions in the submucosa, followed by ringing artifacts (Fig. 1b). We suspected that the patient had pneumatosis intestinalis based on the EUS findings.

**Fig. 2:**
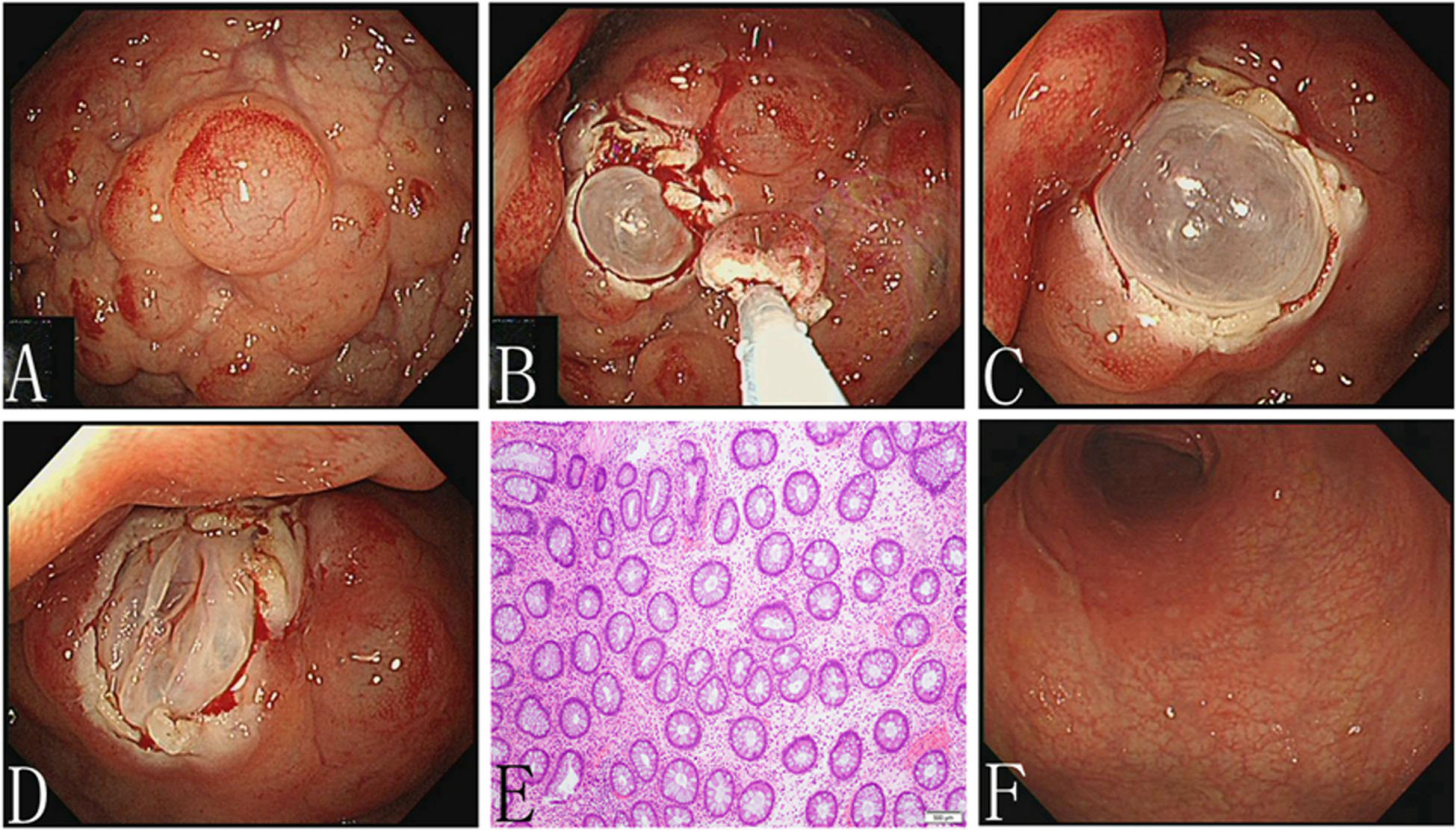
Imaging and pathological features. **a** Cystic nodules were seen, with mucosal hyperemia and erosion. **b** Image after mucosal surface resection. **c** White bubble-like lesions of submucosa were seen. **d** Cyst collapse after fine needle puncture. **e** Pathology revealed chronic inflammation. **f** Colonoscopy showed that the surface of the mucosa was smooth

We performed further examinations to confirm the diagnosis. Intestinal computed tomography (CT) scan showed intramural gas in the sigmoid colon only (Fig. 1c, d, e). Consequently, the diagnosis of PCI was gradually established. To obtain better management, we used forceps to break the sac wall to exhaust the gas. The white bulge of the submucosa resembled a bubble after we used a snare to resect the surface of the mucosa with high-frequency electroscission (Fig. 2a, b, c). After being punctured by needles, the bubbles collapsed and the mucosa was removed and sent for pathological examination (Fig. 2d). Chronic mucosal inflammation was noted (Fig. 2e). The patient’s symptoms resolved, and he was cured 3 months later after administering intestinal flora microecological therapy and other conservative medical treatments (Fig. 2f). In addition to administering 5-aminosalicylic acid, we recommended that the patient discontinue the use of alpha-glucosidase inhibitor (α-GI) in the follow-up. No recurrence of digestive tract symptoms or other discomfort occurred during the 1 year of follow-up.

## Discussion and conclusions

PCI can be divided into primary (15%) and secondary (85%) types [2, 4]. The secondary type occurs secondary to diseases such as digestive tract stenosis, obstructive pulmonary disease, abdominal external injury or surgery, and malnutrition [2, 3]. There are three hypotheses of PCI pathogenesis: (1) mechanical theory: involving an increase in intraluminal pressure that causes mechanical damage and mucosal rupture of the intestinal wall, leading to the migration of gas from the gastrointestinal cavity to the intestinal wall [1]; (2) pulmonary theory: chronic lung diseases such as chronic obstructive pulmonary disease, asthma, and interstitial pneumonia lead to alveolar rupture, causing mediastinal emphysema and release of gas along the aorta and mesenteric blood vessels into the intestinal wall [5]; and (3) bacterial theory: aerogenic bacteria penetrate the intestinal mucosal barrier, ferment in the intestinal wall, and produce gas [6].

In the present case, the patient was taking an alpha-glucosidase inhibitor (α-GI) to control blood glucose. Some scholars reported that α-GI use was associated with the pathogenesis of PCI [7, 8]; α-GIs suppress the absorption of carbohydrates by inhibiting α-GI activity. Carbon dioxide, hydrogen, methane, and other metabolites are produced by the fermentation of carbohydrates. Meanwhile, pneumocysts are formed due to increased intraluminal pressure, which is attributed to peristaltic hypofunction associated with diabetes mellitus and gas-producing bacteria breaking through the mucosal integrity and invading the mucosa. Patients improve with conservative treatment, such as fasting, fluid replacement, and discontinuation of α-GIs. Since about 30% of Japanese diabetic patients use α-GIs, recent reports on the relationship between α-GIs and PCI have been published primarily in Japan [7]. Kojima reported a case of PCI associated with miglitol [9]. However, whether the etiology of PCI is related to α-GIs requires further exploration. Therefore, in clinical practice, when patients with diabetes complain of gastrointestinal symptoms, the possibility of PCI should be considered.

PCI lesions are mainly located in the colon (46%) and small intestine (27%), followed by the large and small intestine (7%) and stomach (5%) [10]. The clinical manifestations of primary PCI are nonspecific, such as abdominal pain (59%), diarrhea (53%), nausea and vomiting (14%), mucus in stool (12%), and hematochezia (12%) [2]. Secondary PCI also has primary disease manifestations. About 3% of the patients with PCI complained of complications, including pneumoperitoneum, volvulus, intestinal obstruction, and intestinal ischemia [1, 2, 11–13]. Serious complications may alter the decision-making process for the therapeutic schedule.

The laboratory examination and pathological biopsy of PCI are nonspecific; the diagnosis mainly depends on colonoscopy, CT, radiography, and ultrasound findings. However, it is easily confused with intestinal polyps [14], cancer [15], or inflammatory bowel disease [16], necrotising enterocolitis [17], even if colonoscopy and biopsy are performed due to a lack of awareness of PCI. Many cases can be found in clinical practice in which patients with pneumoperitoneum signs were initially misdiagnosed as having digestive tract perforation and suffered from unnecessary surgery [18–20].

Some researchers reported that the abdominal CT characteristics of PCI are multiple submucosal or subserosal cystic transmission areas that resemble a bunch of grapes [12]. Furthermore, CT images taken at different levels enable estimation of lesion location and extent through three-dimensional scanning. The image can be observed more clearly when adjusted to the lung window. The discovery of the presence of gas in the portal vein is one of the advantages of CT examination, which is particularly important for making therapeutic decisions. Lassandro et al. found that gas in the portal vein was present in approximately 25.5% of PCI patients. In these patients, the incidence of intestinal obstruction increased and the mortality rate increased to 50% [21]. EUS has high diagnostic value in PCI: multiple cystic lesions without echoes are visible at the sigmoid colon with rear ringing artifacts. EUS provides reliable imaging evidence for making a definitive diagnosis. Ribaldone et al. mentioned that EUS is effective for analyzing the nature and source of intestinal masses, which is a unique advantage [22].

Although the diagnosis of PCI mainly depends on medical imaging, biopsy is still highly recommended. First, the endoscopic manifestations of PCI patients usually present as polypoid or protuberant lesions. With the development of industrialized countries in the twenty-first century, the incidence of Crohn’s disease and colon cancer has continually increased and concomitantly, the number of colonoscopy examinations [23, 24]. The symptoms, location, and endoscopic manifestations of PCI are also similar to those of other diseases, such as inflammatory bowel disease, intestinal neoplasms, and intestinal polyps [14–16]. Additionally, PCI has been associated with Crohn’s disease and both can coexist in the same patient [25]. Moreover, the specific etiology of Crohn’s disease combined with PCI is unclear, which may be a result of the surgical history of Crohn’s disease patients [26]. A pathological biopsy cannot be neglected in this situation and can be conducive for accurate and systematic analysis. Pathological findings of giant cell arrays and partial or collapsed cysts may be helpful in differentiating PCI [27]. Therefore, increasing the doctor’s understanding of PCI is imperative for proper management. Making correct judgments on PCI avoids increasing the psychological and economic burden on patients and unnecessary medical procedures, such as mucosal dissection and surgery, especially by doctors in grassroots units, whose clinical experience is relatively lacking.

If a PCI is suspected, CT examination and ultrasound endoscopy should be performed when conditions permit, and the factors causing PCI should be explored. In addition to gastrointestinal diseases and emphysema, some rare events are associated to PCI, such as alpha-glucosidase inhibitors [7], sunitinib [20], lung transplantation [28], bone marrow transplantation [29], systemic lupus erythematosus [30], systemic sclerosis [11], myeloma [31], granulomatosis with polyangiitis [32]. Moreover, the mucosal damage caused by colonoscopy and biopsy may result in the gas entering the intestinal mucosa, thereby promoting the occurrence of PCI [33]. Therefore, it is not enough to reach a superficial diagnosis, as more examination are necessary to investigate the disease etiology. In our case, the cause of PCI could be related to the use of α-GI. It is worth mentioning that in 2019 we re-enrolled a patient with PCI who also had a history of taking acarbose, who is currently undergoing treatment. For patients with PCI, we should investigate their medical history as far as possible, inquring about the recent use of alpha-glucosidase inhibitor, multi-tyrosine kinase inhibitor, glucocorticoid, and other drugs, exploring whether there is a combination of lung diseases, gastrointestinal diseases, diabetes, autoimmune diseases, cancer history, and organ transplantation history. At the same time, combined with medical history is beneficial to pertinently screen the pathogeny and eliminate potential influencing factors, such as mucosal damage, increased intestinal pressure, and bacterial infection. A thorough assessment of the clinical background of PCI and the correct comprehension of the differential diagnosis of PCI is essential to avoid unnecessary surgery.

Most researchers believe that PCI is a benign disease with conservative treatments: (1) observation; (2) oxygen or hyperbaric oxygen therapy. Kensuke Nakatani et al. reported that hyperbaric oxygen therapy is the preferred method [32]; (3) antibiotics including metronidazole and quinolones can inhibit intestinal bacterial infection; and (4) endoscopic treatment. Endoscopic fine needle aspiration contributes to the diagnosis and treatment of PCI, by puncturing the cyst to exhaust gas [34–37]. PCI-induced intestinal obstruction can be treated by the high-freguency endoscopic resection of the cyst wall, and cyst collapse after gas discharge [36]. Because the presence of multiple sites of operations and biopsies increase the risk of infection by nearly 9 times, local implementations are generally recommended [36, 37]. However, some patients developed intestinal obstruction again after relieving the first obstruction. At this point, sclerotherapy of the cyst wall after puncture may be a solution to prevent the cyst from expanding again [18].

Surgical intervention is not absolutely necessary to treat PCI. How accurately identifying the timing of surgery has become a clinical challenge. Generally speaking, the prognosis of PCI is good, and the poor predictors of prognosis include pH value < 7.3, bicarbonate level < 20 ml/L, lactate level > 2 mmol/L, amylase level > 200 U/L, and presence of portal venous gas [4, 11, 13, 38]. A level of > 2 mmol/l of lactate was the strongest predictor of pathological PCI and correlated with adverse outcomes [13]. In order to more rapidly identify and manage patients with severe PCI, patients should be divided into three groups: patients requiring surgery, patients with invalid surgery, and patients with benign intestinal emphysema. The primary operative indications are considered if the patient meets any of the following criteria: obstructive symptoms, WBC > 12 c/mm^3^ or CT findings of portal vein gas, especially when the patient is older than 60 years old, due to the high mortality rate associated with this condition. For secondary indications, if patients have sepsis or signs of acidosis (pH < 7.3, lactate > 2.0, bicarbonate < 20), surgery should be performed [4]. Patients with acute abdomen, acute kidney injury, or hypotension can also be treated surgically [38]. Accordingly, PCI patients should undergo detailed physical examination and as well as renal function and blood-gas analysis, CT, and tests for amylase and C-reactive protein levels, along with other tests, so as to quickly identify patients who need surgery and patients who can be relieved by conservative treatment. Although lactic acid was not detected in our case, the patient was evaluated for benign PCI from abdominal signs, white blood cells, blood pressure, and CT. Therefore, non-surgical treatments to eliminate cysts were selected. PCI is relatively unfamiliar to inexperienced clinicians, and an optimal PCI management model has yet to be suggested.

To conclude, PCI is a not uncommon disease with male predominance and unclear etiology and pathogenesis. Because abdominal pain, diarrhea, and other nonspecific abdominal symptoms are the main clinical manifestations, it is easily confused with intestinal polyps, cancer, or inflammatory bowel disease. With increase in the number of endoscopies, it is essential to improve the doctor’s comprehension of PCI. The diagnosis mainly depends on abdominal CT and colonoscopy findings. For example, CT shows a number of grape-like or beaded low-density cystic light transmission areas. Treatment includes observation, oxygen therapy, endoscopic treatment, and surgery. The treatment should be tailored to the clinical symptoms and endoscopic manifestations to avoid unnecessary surgery. If no serious complications occur, the prognosis is optimistic.

## Data Availability

All information about the patient come from department of Gastroenterology, Wuhan Union Hospital. The data used and analyzed during the current study are included in this article.
